# Developmental Trajectories of Amygdala and Hippocampus from Infancy to Early Adulthood in Healthy Individuals

**DOI:** 10.1371/journal.pone.0046970

**Published:** 2012-10-09

**Authors:** Akiko Uematsu, Mie Matsui, Chiaki Tanaka, Tsutomu Takahashi, Kyo Noguchi, Michio Suzuki, Hisao Nishijo

**Affiliations:** 1 Department of Psychology, Graduate School of Medicine and Pharmaceutical Sciences, University of Toyama, Toyama, Japan; 2 Department of Pediatrics, Graduate School of Medicine and Pharmaceutical Sciences, University of Toyama, Toyama, Japan; 3 Department of Neuropsychiatry, Graduate School of Medicine and Pharmaceutical Sciences, University of Toyama, Toyama, Japan; 4 Department of Radiology, Graduate School of Medicine and Pharmaceutical Sciences, University of Toyama, Toyama, Japan; 5 Department of System Emotional Science, Graduate School of Medicine and Pharmaceutical Sciences, University of Toyama, Toyama, Japan; George Mason University/Krasnow Institute for Advanced Study, United States of America

## Abstract

Knowledge of amygdalar and hippocampal development as they pertain to sex differences and laterality would help to understand not only brain development but also the relationship between brain volume and brain functions. However, few studies investigated development of these two regions, especially during infancy. The purpose of this study was to examine typical volumetric trajectories of amygdala and hippocampus from infancy to early adulthood by predicting sexual dimorphism and laterality. We performed a cross-sectional morphometric MRI study of amygdalar and hippocampal growth from 1 month to 25 years old, using 109 healthy individuals. The findings indicated significant non-linear age-related volume changes, especially during the first few years of life, in both the amygdala and hippocampus regardless of sex. The peak ages of amygdalar and hippocampal volumes came at the timing of preadolescence (9–11 years old). The female amygdala reached its peak age about one year and a half earlier than the male amygdala did. In addition, its rate of growth change decreased earlier in the females. Furthermore, both females and males displayed rightward laterality in the hippocampus, but only the males in the amygdala. The robust growth of the amygdala and hippocampus during infancy highlight the importance of this period for neural and functional development. The sex differences and laterality during development of these two regions suggest that sex-related factors such as sex hormones and functional laterality might affect brain development.

## Introduction

In the past two decades, morphometric brain differences among different phenotypic groups have become evident due to the availability of a non-invasive brain imaging method, magnetic resonance imaging (MRI) [1], [2]. Although most of these studies compared certain groups at one point in time rather than across time, some studies emphasized the importance of comparing developmental trajectories among the different groups rather than merely comparing the average volumes at one point of time [3], [4]. These studies reported that developmental trajectories were different as a function of sex [5], IQ [6], premature birth [7], and neuropsychiatric disorders [8]. Hence, knowledge of developmental trajectories of the human brain may help to understand how its neural and functional development will progress after birth.

However, most of these brain development studies did not include infant data. Even those that did include such data measured only total brain volume, volumes in four of the cortical lobes, and/or gray and white matter in the whole brain [3], [9]. Although one group [10] did include normal infants, to date, they have not reported volumetric data based on their MRI. Likewise, most of the previous studies did not measure subcortical brain regions such as the amygdala and hippocampus. The amygdala and hippocampus constitute major components of the limbic system and are implicated in emotion and memory, which are indispensable brain functions from the beginning of life. Furthermore, volume changes in the amygdala and hippocampus were often reported in patients with developmental disorders such as autism spectrum and schizophrenia [11], [12]. Schumann [11] reported children with autism spectrum had bigger amygdala size at the age of 8 than healthy children, but their amygdale volume did not change with age although that of healthy children grew with age. Thus, knowledge of typical morphological development of the amygdala as well as the hippocampus would contribute to understanding brain functional development.

Nonetheless, the typical development of the amygdala and the hippocampus remains unclear, because few studies have measured these over the life span, following the infancy. Giedd [1] based on a male only sample, reported an increase in amygdala volume between 4 to 18 years of age. Mosconi et al [12] compared the developmental trajectory of the amygdala between autistic and non-autistic children from 18 to 35 months and reported no difference of increase in amygdale volume over time between the two groups, although there were size differences between the groups (autistic children > non-austistic children). Brenhouse and Andersen [13] in a review concluded that the female amygdala reached its maximal volume by the age of 4 and that only a small increase of its gray matter volume could be seen during adolescence, whereas the volume of the male amygdala increased by 53% between 4 and 18 years of age. Nonetheless, only a few studies have reported on the amygdala volume of healthy children between 0 and 4 years of age.

Regarding the development of the hippocampus, Knickmeyer et al [14] reported a 13% increase of hippocampal volume from one to two years of age (but relatively little growth could be seen after it was normalized for total brain volume). Giedd el al [1] reported that the right hippocampus correlated with age only in females, and that the left hippocampus did not increase with age between 4 to 18 years in males, or females. Although each of these studies have compensated for the lack of developmental periods since the age of participants in each study has been during a defined period, it is difficult to combine them and obtain an accurate picture of the complete longitudinal developmental trajectory of the hippocampus and the amygdala over the age span, because of the methodological differences between the different studies.

Some studies have reported sexual dimorphisms in the amygdala and hippocampus [1], [13], and based on animal studies these two regions are also known to have many receptors for sex hormones such as estrogen and androgen [15], [16]. Some recent studies have shown hormonal effects on human brain development [17]–[20]. Lombardo et al. [17] showed that increased fetal testosterone is predictive of a ventral medial subregion in the left amygdala. Buss et al. [18] found that increased maternal cortisol levels during the early gestational period were substantially associated with larger right amygdala volumes among girls. Neufang et al. [19] examined the relationships between steroid levels, pubertal stages, and brain structure in the sexually dimorphic regions, and found that gray matter volume in the amygdala is predicted by testosterone levels in both males and females, with testosterone levels also predictive of hippocampal size in females. Bramen et al. [20] observed significant interactions between sex and the effects of puberty for brain regions with high sex steroid hormone receptor densities; sex differences in the right hippocampus and bilateral amygdala were more pronounced in more sexually mature adolescents. Specifically, larger hippocampus and amygdala volumes were observed in more sexually mature boys, whereas smaller volumes were observed in more sexually mature girls. In addition, although previous studies examined laterality of the amygdala and hippocampus, findings have been inconsistent across such studies [21]. Thompson et al. [7] suggested that laterality might depend on the period of development, gestational age at birth, and mental health. Thompson el al. [7] also studied laterality of the infant hippocampus (pre- and full-term), and found that the right hippocampus was significantly larger than the left in infants, suggesting that infants have greater need for right hippocampus functions such as visuo-spatial abilities, as compared to left hippocampus functions like linguistic ability. These studies imply that both sex and laterality can influence the development of the amygdala and hippocampus.

Taken together, the purpose of this study was to examine typical volumetric trajectories of the amygdala and hippocampus, as well as intracranial volume and the whole brain, from infancy to early adulthood. This would clarify sexual dimorphism and laterality during development of these brain regions.

## Methods

### Participants

The MRI data were collected from 1998 to 2010. Participants included 111 healthy and normally developing Japanese (58 males and 53 females) from 1 month to 25 years old (mean monthly age ± S.D. = 108.75±83.71). The age and gender distribution for age brackets were provided in Table 1. The participants below the age of 18 were children of University hospital staff, of parents attending a community parenting class, or in schools in Toyama city. All the children were born full-term (gestational age ranged between 37–41 weeks). Participants above 18 years old were students recruited from Toyama Medical and Pharmacological University. All were screened with a health questionnaire through interviews, which found no evidence of health issues or abnormal neurological development. Their heights and weights were all within the normal range, and all were right-handed. After the purpose and procedures of the study were fully explained, written informed consent was obtained from the participants, and/or the participants’ parents if the participants were below 18 years old. This study was reviewed and approved by the Research and Ethics Committees at the University of Toyama. One scan had very large artifacts due to head movement, so a total of 109 images (57 males and 52 females) were obtained.

**Table 1:** Frequency of participants.

Age(years)	Male	Female	Total
0–1	5	10	15
1–2	4	7	11
2–4	8	3	11
4–6	4	3	7
6–8	2	3	5
8–10	4	7	11
10–12	4	4	8
12–14	4	6	10
14–16	3	5	8
16–18	4	4	8
18–20	5	4	9
20–25	2	4	6
Total	57	52	109

### Imaging Acquisition

T1-weighted axial images were obtained on 1.5 T Magnetom Vision scanner (Siemens, Erlangen, Germany), using the fast low angle shot gradient refocused 3-dimensional sequence with the following parameters: echo time (TE) = 6 ms, repetition time (TR) = 35 ms, flip angle = 35°, nex = 1, field of view = 256 mm, and matrix size = 256×256. The entire scan was completed in 15 minutes. We obtained 140 to 180 contiguous slices from each participants scanned on the same scanner, with each slice having a thickness of 1.0 mm. Before the MRI scanning, 47 participants below the age of six years were sedated with oral monosodium trichorethyl phosphate syrup (0.5–1.0 ml/kg), which is routinely used in clinical situations and has demonstrated safety. This drug was administered once only. One child that did not fall asleep after the single dose was discontinued from the study.

### Image Analysis

For intracranial volume and total brain volume, the images were transferred to a Linux workstation. Image processing was performed with Dr. View image analysis software (Asahi Kasei Joho System Co., Ltd., Tokyo, Japan). The intracranial volume (ICV) was manually traced based on Whitewell et al [22]. This manual tracing was preceded in axial view from superior to inferior, by drawing around the dura, excluding any bone marrow or any non-brain but including the superior sagittal sinus. Optic nerves were also excluded. The whole brain volume was measured based on Matsuzawa et al [9]. Total brain volume (TBV) was defined by the brain region without CSF, which was masked with similar procedures for ICV using Dr. View. The caudal brain stem regions below the level of the cerebral peduncles of the midbrain were excluded. The hypothalamic and chiasmatic cisternae were retained, but the pituitary, the carotid cisterna, the ambient cisterna and the quadrigeminal plates were excluded.

**Figure 1:**
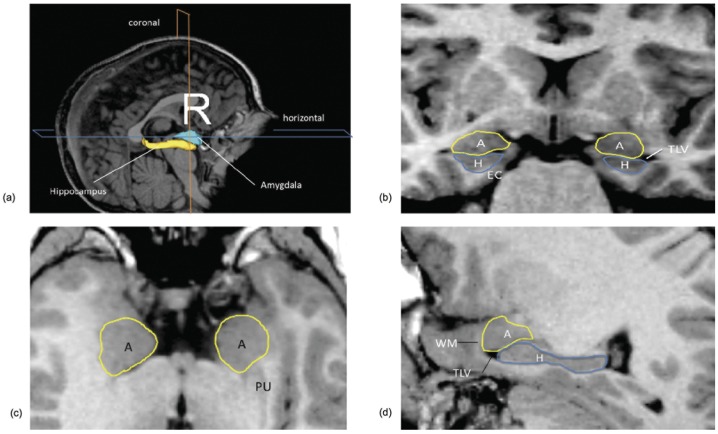
Orthogonal views for segmenting the amygdala and hippocampus on MRI sections. A three-dimensional reconstruction of images (a) in which lines indicate the position of the horizontal plane (b), sagittal plane (c), and coronal plane (d) is shown. A, Amygdala; EC, entorhinal cortex; H, hippocampus; PU, putamen; TLV, temporal horn of the lateral ventricle; WM, subamygdaloid white matter.

For the amygdala and hippocampus volumes, the images were imported into the 3D-Slicer software (http://www.slicer.org/) and manually defined as previously described in Schumann et al [11]. Briefly, images were reoriented along the horizontal axis from the rostral to the caudal pole of the hippocampus to aid in distinguishing the amygdala from the hippocampus (Figure 1a). The initial amygdala and hippocampus tracing process involved defining the borders in coronal sections, starting with the most caudal level of the hippocampus (where the fornix was visible) to the approximately most rostral section in which the amygdala was present. The boundaries between the amygdala and hippocampus are the lateral ventricle or the alveus of the hippocampus (Figure 1b). Outlines were then verified and edited in the axial and sagittal views that were simultaneously available to the rater while tracing (Figure 1c and 1d).

Inter-rater reliability was examined in a sample of 20 randomly selected brain scans (thirteen infants or children and seven adolescents or adults, male vs. female = 10∶10), as analyzed by two raters (A.U. and M.M.T.). The intraclass correlation (ICC) for the amygdala was.84, and that for the hippocampus was.80. The ICCs for only infants/children were.82 for the amygdala and.85 for the hippocampus, and those for only adolescents/adults were.93 for the amygdala and.80 for the hippocampus. ICCs for males were.81 for the amygdala and.80 for the hippocampus, and those for females were.88 for the amygdala and.80 for the hippocampus. The rater A.U. then completed the analysis on the remaining scans.

### Statistical Analysis

With SPSS 19.0, *F* tests in regression analyses of linear, quadratic, and cubic models were used to determine whether age (in months) is related to volume changes of the whole brain, amygdala, and hippocampus, dividing the sample into the female and the male group. The *R^2^* values were used to decide which linear or nonlinear models best characterized the development of each region. From the estimated models, the peak ages and the local maximal volumes of each given region were calculated for the whole, female, and male groups, if possible. In addition, from the best characterized model, the differentiation ( = growth change) in each month was calculated and graphed from 1 month until the peak age.

Analysis of variance (ANOVA) was performed to test the effects of sex and age group for each volume.

To examine laterality, right and left amygdala and hippocampus volumes were expressed as a laterality index as:
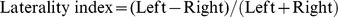



A one-sample *t* test was performed for each subsample (testing against the reference value of 0.00). In addition, a linear regression analysis was performed to reveal any relationship between laterality and age.

In addition to the raw measured volumes, we also examined the volumes adjusted by ICV, using the following equation [23]:
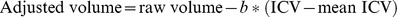
where *b* is the slope of a regression line of a region of interest volume on ICV. Mean ICV was calculated separately by each year of age for ages below 15 because the volume increase year by year was enormous. Mean ICV was calculated as a single mean for participants above the age of 16.

We defined statistical differences at the 5% level as being significant. To prevent a possible Type I error due to multiple tests, a Bonferroni correction was applied for correlation analyses.

## Results

**Figure 2:**
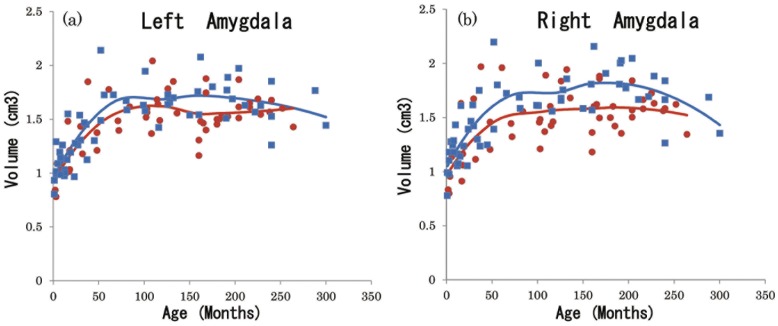
Scatter plots by monthly age and sex of absolute left and right amygdalar volume with locally weighted scatter plot smoothness (LOWESS). (a); LA: left amygdala, (b); RA: right amygdala, blue square: male (n = 57), red circle: female (n = 52), blue line: males’ LOWESS, red line: females’ LOWESS.

**Figure 3:**
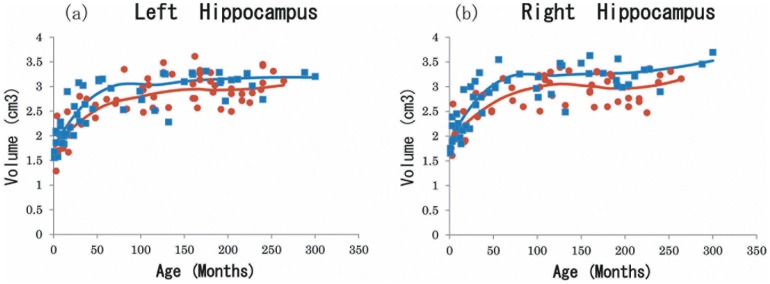
Scatterplots by monthly age and sex of absolute left and right hippocampal volume with locally weighted scatterplot smoothness (LOWESS). (a); LH: left hippocampus, (b); RH: right hippocampus, blue square: male (n = 57), red circle: female (n = 52), blue line: males’ LOWESS, red line: females’ LOWESS.

Scatter plots representing the amygdale and hippocampi are illustrated in Figs. 2 and 3 with locally weighted scatter plot smoothness (LOWESS). A LOWESS curve allows the form of a curve to be identified by using actual data. Therefore, this approach is useful for identifying data patterns that may otherwise be overlooked by using curve-fitting procedures that assume a specific shape [24].

### Estimated Curves in the Whole Brain, Amygdala, and Hippocampus

The regression analyses revealed that monthly ages were significantly related to volume changes in the whole brain, amygdala, and hippocampus in all of the linear, quadratic, and cubic models (all *p<*.001). The *R* ^2^ values were highest in the cubic model for every region: whole brain [F(3,87) = 41.09, *p*<.001], left amygdala (LA) [F(3,87) = 22.85, *p*<.001], right amygdala (RA) [F(3,87) = 16.05, *p*<.001], left hippocampus (LH) [F(3,87) = 33.64, *p*<.001], and right hippocampus (RH) [F(3,87) = 35.98, *p*<.001]. These results were consistent after adjustment and division by sex (Table 2).

**Table 2:** Best fitting developmental trajectory model with peak ages.

Brain Region		Best Fitting Model	Sex	R^2^	Age of Peak
Whole Brain		Cubic	Whole	0.61	9.3
			Male	0.72	9.8
			Female	0.60	8.4
Left Amygdala		Cubic	Whole	0.60	10.6
			Male	0.67	11.1
			Female	0.55	9.6
Right Amygdala		Cubic	Whole	0.48	11.8
			Male	0.64	12.6
			Female	0.41	11.4
Left Hippocampus		Cubic	Whole	0.66	11.2
			Male	0.70	11.4
			Female	0.67	11.0
Right Hippocampus		Cubic	Whole	0.64	9.8
			Male	0.73	10.3
			Female	0.61	9.7
Adjusted Whole Brain		Cubic	Whole	0.77	9.8
			Male	0.75	9.5
			Female	0.81	9.5
Adjusted Left Amygdala		Cubic	Whole	0.60	10.7
			Male	0.57	10.9
			Female	0.63	9.4
Adjusted Right Amygdala		Cubic	Whole	0.52	12.2
			Male	0.54	12.8
			Female	0.51	11.4
Adjusted Left Hippocampus		Cubic	Whole	0.63	11.2
			Male	0.63	10.9
			Female	0.64	10.7
Adjusted Right Hippocampus		Cubic	Whole	0.63	9.9
			Male	0.69	10.0
			Female	0.58	9.9

### Peak Ages and Growth Changes

Table 2 shows the peak ages calculated using the estimated cubic models. The female group reached its local maximal volume earlier than the male group in all regions. In addition, the left amygdala (LA) reached its peak around 1.5 to 2 years earlier than the right amygdala (RA) did, whereas the left hippocampus (LH) reached its peak around 1 year later than the right hippocampus (RH) did.

Table 3 shows mean volumes of the whole brain, amygdala and hippocampus by sex in infants (age <2 years), children (2 years < age <10 years) and adolescents/adults (age >10 years). Given that our earlier work found evidence for a peak growth spurt at around two years of age [9], boys and girls aged 1 to 24 months were included in the Infant category. Adolescents/adults were categorized as aged 10 years or older, on the basis of the present results showing peak brain volume at around this age. ANOVAs (sex×age group) for whole brain volume, amygdala volume and hippocampal volume showed main effects of sex (whole brain: males > females, F(1, 103) = 4.72, *p = *0.03; amygdala: males > females, F(1, 103) = 4.78, *p = *0.03; hippocampus: males > females, F(1, 103) = 8.11, *p = *0.005) and age group (whole brain: F(2, 103) = 56.20, *p*<0.001; amygdala: F(2, 103) = 61.70, *p*<0.001; hippocampus: F(2, 103) = 106.25, *p*<0.001).

**Table 3:** Mean volume of whole brain, amygdala and hippocampus (cm^3^).

	Infants		Children		Adolescents/Adults	
Sex	Male	Female	Male	Female	Male	Female
n	18	9	17	17	22	26
Whole Brain	781.5 (208.9)	753.6 (185.0)	1113.0 (118.1)	1058.2 (74.1)	1197.0 (107.2)	1086.1 (108.2)
Amygdala	2.28 (0.35)	2.12 (0.45)	3.15 (0.50)	3.04 (0.45)	3.43 (0.38)	3.16 (0.34)
Hippocampus	4.24 (0.67)	3.98 (0.72)	5.80 (0.58)	5.57 (0.51)	6.46 (0.54)	5.94 (0.52)

note: Infants are whithin 1 to 24 months. Childeren are above 24 months to 10 years old.

Adolescents/adults are above 10 years old. () shows s.d.

**Figure 4:**
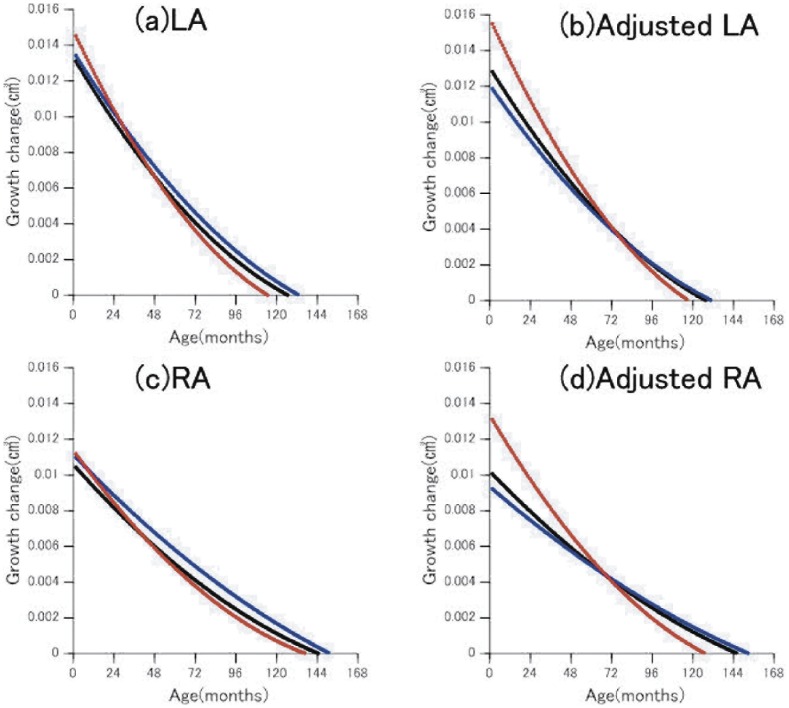
Rate of growth change in amygdalar volumes. (a); LA: left amygdala, (b);left amygdala adjusted by ICV, (c); RA: right amygdala, (d);right amygdala adjusted by ICV. The rates of monthly volume changes in the right and left amygdala. Positive values indicate increasing volume. The point of intersection on the x-axis represents the age of local maximal volume. Black line: whole group, blue line: males, red line: females.

**Figure 5:**
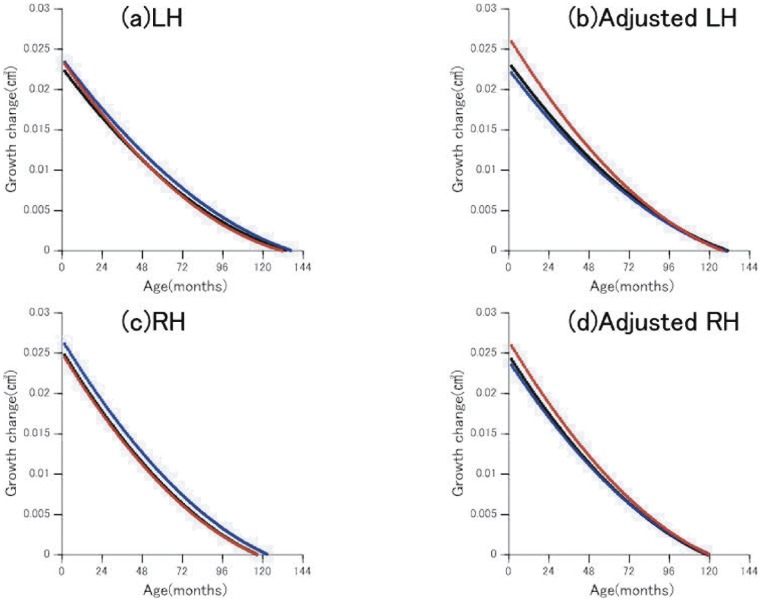
Rate of growth change in hippocampal volumes. (a); LH: left hippocampus, (b);left hippocampus adjusted by ICV, (c); RH: right hippocampus, (d);right hippocampus adjusted by ICV. The rates of monthly volume changes in the right and left hippocampus. Positive values indicate increasing volume. The point of intersection on the x-axis represents the age of local maximal volume. Black line: whole group, blue line: males, red line: females.

Figures 4 and 5 depict growth changes of the amygdala and hippocampus until the peak age, all of which showed a rapid volume increase in the first few months from birth regardless of sex. The female LA growth change was larger for the first few years when adjusted, but its rate decreased earlier than that of the males (Fig. 4). The growth change of the RA was smaller than that of the LA, but its sexual difference trend was similar (Fig. 4). After peak age both LA and RA volumes for males were significantly larger than those for females (LA, male 1.68±0.19, female 1.56±0.17, t(43) = 2.15, *p = *0.037; RA, male 1.76±0.23, female 1.57±0.17, t(39) = 3.14, *p = *0.003), while before peak age there were no significant sex differences for either LA or RA (LA, male 1.34±0.31, female 1.39±0.32, t(62) = 0.60, *p = *0.550; RA, male 1.42±0.31, female 1.40±0.34, t(66) = 0.22, *p = *0.83).

In the hippocampus, the female and male groups revealed similar curves in raw LH and RH, but after adjustment the females showed a greater increase during the first several years (Fig. 5). After peak age, both LH and RH volumes for males were significantly larger than those for females (LH, male 3.17±0.21, female 2.95±0.25, t(40) = 3.09, *p = *0.003; RH, male 3.30±0.29, female 3.00±0.30, t(46) = 3.61, *p*<0.001), while before peak age there were no significant sex differences for either region (LH, male 2.48±0.55, female 2.46±0.49, t(65) = 0.15, *p = *0.883; RH, male 2.59±0.52, female 2.60±0.49, t(59) = 0.09, *p = *0.93).

The growth rate change of the whole brain volume also showed a large increase during the first several years of life, and the males displayed a larger growth change across time than the females did. However, after adjustment by ICV, these differences became less obvious.

### Laterality

The hippocampus displayed significant rightward volumetric asymmetry in both males and females, which remained after adjustment by ICV (laterality index of male: Mean±S.D. = −.031±.037, t (56) = −6.385, *p* = .000; laterality index of female: Mean±S.D. = −.022±.038, t(51) = −4.189, *p* = .000). There was no significant hippocampal laterality index difference between males and females, t(107) = 1.234, *p = *.220. However, significant asymmetry (rightward) of the amygdala was observed only in the males, an asymmetry that persisted after adjustment by ICV (laterality index of male: Mean ±S.D. = −.023±.037, t(56) = −4.734, *p* = .000; laterality index of female: Mean±S.D. = .0007±.038, t(51) = 0.141, p = .888). The amygdala laterality index was higher in males than females, t(107) = 3.358, *p = *.001.

**Figure 6:**
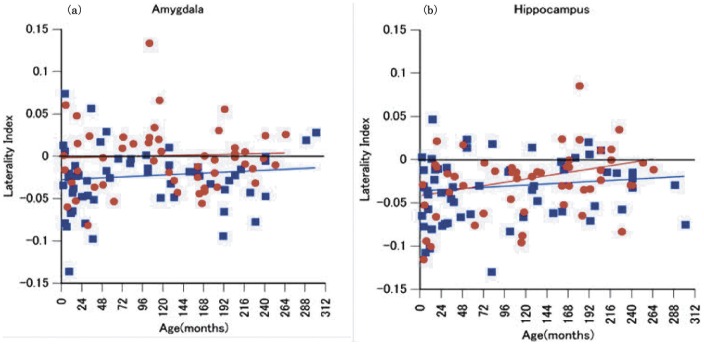
The relationship between laterality index and age. (a) amygdala, (b) hippocampus. Blue square and blue line: male. Red circle and red line: female.

Figure 6 shows the relationship between age and laterality index. The linear regression analysis for age and laterality index indicated that only the female laterality index of the hippocampus significantly changed – from negative to 0 (rightward to symmetrical) as a function of age [F(1,50) = 5.930, *p* = .018], but not that of male [F(1,55) = 1.044, *p* = .311]. However, correlations were not statistically different across males and females (males: r = .136, females: r = .326, z = 1.021, *p = *.307). In amygdale, there were no significant correlations between age and laterality index in both male [F(1,55) = .660, *p* = .420] and female [F(1,50) = .139, *p* = .710. Again, correlations did not differ across males and females (males: r = .109, females: r = .053, z = .286, *p = *.775). After adjustment of volumes by ICV, the above all correlations were similar [male adjusted hippocaumpus r = .119, F(1,55) = .785 *p* = .380, female adjusted hippocaumpus r = .321, F(1,50) = 5.758, *p* = .020, z = 1.081, *p* = .280; male adjusted amygdala r = .129, F(1,55) = .934, *p* = .338, female adjusted amygdala r = .055, F(1,50) = .152, *p* = .698, z = .378, *p* = 705].

## Discussion

The volumes of the whole brain, amygdale and hippocampi exhibited significant age-related changes from infancy to early adulthood, even after adjustment by ICV. The cubic models best characterized the estimated developmental trajectories of all the given brain regions, regardless of sex, or hemispheric differences. For the whole brain, the cubic model was consistent with a previous study by Lenroot, et al [5]. However, to our knowledge, no previous study has estimated the non-linear developmental models of the amygdala of healthy humans from infancy to early adulthood. Giedd [1] reported no volumetric changes of amygdala in females between 4 and 18 years of age. The results of the present study indicated that the maximum volume of the amygdala was reached between 9 and 11 years of age. Moreover, the study showed a non-linear, age-related increase in development after 4 years in both males and females. This result, which is inconsistent with Giedd et al [1] might have been caused by the inclusion of data from infants and toddlers, who showed a robust increase in amygdalar volume during the period of the present study. Mosconi et al. [12] reported that amygdalar volumes increased linearly over time in children from 1 to 6 years of age, without regard to any developmental disorders (such as autistic spectrum, or developmental delays). The present findings appear to bridge the gap between the results of these two previous studies [1], [12].

The hippocampus also showed a non-linear developmental pattern, with volume increases in both hemispheres until approximately 9 to 11 years of age. This result was not consistent with previous findings in 4 to 18 year olds). However, other studies have reported volumetric changes in hippocampal sub-regions during this age [25]. Suzuki et al. [26] also demonstrated a significant volume increase in the hippocampus between 13–14 and 18–21 years of age in males. Moreover, a postmortem study by Benes [27] showed that myelination in the subicular and presubicular regions of the hippocampus continued until adulthood. Furthermore, animal studies [28] have shown that the hippocampus is a region where neurogenesis occurs until adulthood. The results of the current study corroborate these previous findings. Complex interactions among genetic factors, environmental conditions, as well as changes in these factors, strongly contribute to volume changes in subcomponents of the brain [29]. Such complex interactions might result in large individual variations in amygdalar and hippocampal development causing statistically inconsistent results.

The present results also corroborated previous studies by showing that the peak age of whole brain development occurred earlier in females, than in males, with the exception that the peak age reported in this study happened earlier than has been reported in previously [1], [3], [5]. Our results were also consistent with previous studies in showing that males had larger whole brain volumes than females [1], [3], [5].

Moreover, the results of this study showed that the right and left amygdala tended to be larger in males than in females. Similar to the whole brain, the female amygdala reached its peak volume approximately 18 months earlier than the male amygdala. In addition, changes in the growth rate of the amygdala decreased earlier in females. These findings suggest that the longer growth period of the amygdala might contribute to the larger male amygdala. Animal studies have indicated that the amygdala has rich receptors for male hormones (androgen), which promotes myelinogenesis [16], [30]. Consequently, myelinogenesis might contribute to increases in the volume of the amygdala in males. Interestingly, however, changes in the growth of the amygdala relative to the intracranial volume were larger in females at the beginning of life, indicating that growth factors affecting the amygdala may change as a result of age and sex.

Our results also indicated that the raw hippocampal volumes of males were larger than those of females. Sex differences in changes in absolute hippocampus growth were less obvious compared to those of the amygdala, relative growth changes of the female left hippocampus were larger than those of males in the first years of life. Gogtay et al. [25] also reported that the development of the posterior hippocampal sub-region is more prominent and is more left lateralized in females. Thus, the larger growth change of the left hippocampus in females might be the result of development in posterior hippocampal sub region. Furthermore, only the female hippocampus laterality index changed significantly as a result of age, causing left and right volumes to become more symmetrical as age increased. It has been reported that females tend to use both hemispheres for information processing through the larger commissural systems [21], which might contribute to symmetrizing the bilateral hippocampi, and to refining and developing strong connections, even after the formation of neural networks are completed. Such knowledge about sexual dimorphism could be a helpful to understand psychiatric disorders and diseases with different prevalence rates between males and females [5], [18], [25], [26], [31]–[33]. According to a meta-analysis of hippocampal volumetric studies in schizophrenia by Adriano et al. [31], the presence of the smallest effect for the left hippocampus in studies including only males may be explained a smaller left hemisphere in males as compared to females, likely due to hormonal factors. Larger right amygdala volume at 3 and 4 years of age has been associated with slower acquisition of social and communicative skills and generally poorer psychosocial outcomes at 6 years of age, within a sample of children with autism spectrum disorder [32]. Juranek et al. [33] found an association between a large right amygdala and high levels of anxiety. Buss et al. [18] found that higher cortisol concentrations during pregnancy were associated with larger amygdala volumes in girls, and with more affective problems in girls than in boys. These findings suggest that brain sensitivities to internal or external developmental factors may differ as a function of sex such that sexual dimorphisms result, which produce gender differences in terms of developmental risk for some psychological disorders.

Only males displayed significant right asymmetry, suggesting that androgen may partly contribute to the asymmetry in the volumes of the amygdala [17]. Regarding this, one study showed that fetal testosterone is significantly correlated with gray matter volume in the left amygdala but not the right, among 8–11 years-old boys [17]. In terms of trajectory differences, the right amygdalar volume increased for a longer period, but changes in its growth rate was larger in the left amygdala during early childhood, regardless of sex. Previous studies have reported that the left amygdala responds predominantly to fearful events and faces [34], [35]. This suggests that the left amygdala might grow larger in the beginning of life, because infants and children need to detect danger through the faces of their caregivers.

This study, consistently with certain previous studies, showed that when age was not a consideration, the right hippocampus of both females and males was significantly larger than the left one [1]. Thomson et al. [7] studied hippocampal asymmetry in full-term and pre-term infants and found that all infants had a larger right hippocampus and suggested that this asymmetry might have occurred because visuo-spatial abilities (right brain) were more important for infants than linguistic abilities (left brain). Moreover, some studies have suggested that abnormal hippocampal asymmetry was associated with lower IQ, memory impairments, cognitive deficits, and neuropsychological disorders [7]. These results suggest that hippocampal asymmetry might depend on sex, brain functions required at a given age, the developmental period and environmental factors [7], [21], [36], [37].

Several limitations of this study should be noted. First, the developmental trajectory model was calculated from cross-sectional, instead of longitudinal data. Therefore, the results might have over- or under- estimated the true values. A powerful tool for analyzing brain development is the combination of cross-sectional and longitudinal data [5]. Therefore, it is suggested that this study should be replicated using longitudinal data, so that the combined results could be used to develop a more accurate trajectory of the typical brain developments in healthy individuals. In addition, change in volume, by itself cannot directly show the development of neural networks, which could tell us how functional localization and neural circuits have developed over time. Therefore, using combinations of data from volumetric, psycho-developmental testing; fractional anisotropy and fiber tractography through diffusion tensor imaging studies are needed to expand our understanding of neural development.

### Conclusion

This cross-sectional study demonstrated how amygdale and hippocampi in healthy individuals develop from infancy to early adulthood. The study showed significant age-related changes in the volumes of amygdala and hippocampus. The developmental trajectories of the brain regions were best characterized by the cubic model, which demonstrated robust changes in the beginning of life, with the amygdalar and hippocampal volumes peaking around 9 to 11 years of age, suggesting that infancy and preadolescence are critical periods for neural development. In addition, certain patterns of development in these two regions differed according to the sex and brain hemispheres.

## References

[1] Giedd JN, Vaituzis AC, Hamburger SD, Lange N, Rajapakse JC, et al (1996) Quantitative MRI of the temporal lobe, amygdala, and hippocampus in normal human development: ages 4–18 years. J Comp Neurol 366: 223–230. 8698883 10.1002/(SICI)1096-9861(19960304)366:2<223::AID-CNE3>3.0.CO;2-7

[2] Schumann CM, Barnes CC, Lord C, Courchesne E (2009) Amygdala enlargement in toddlers with autism related to severity of social and communication impairments. Biol Psychiatry 66: 942–949. 19726029 10.1016/j.biopsych.2009.07.007 PMC2795360

[3] Courchesne E, Chisum HJ, Townsend J, Cowles A, Covington J, et al (2000) Normal brain development and aging: quantitative analysis at in vivo MR imaging in healthy volunteers. Radiology 216: 672–682. 10966694 10.1148/radiology.216.3.r00au37672

[4] Giedd JN, Lenroot RK, Shaw P, Lalonde F, Celano M, et al.. (2008) Trajectories of anatomic brain development as a phenotype. Novartis Found Symp 289: 101–112; discussion 112–108, 193–105. 10.1002/9780470751251.ch9 PMC3024856 18497098

[5] Lenroot RK, Gogtay N, Greenstein DK, Wells EM, Wallace GL, et al (2007) Sexual dimorphism of brain developmental trajectories during childhood and adolescence. Neuroimage 36: 1065–1073. 17513132 10.1016/j.neuroimage.2007.03.053 PMC2040300

[6] Shaw P, Greenstein D, Lerch J, Clasen L, Lenroot R, et al (2006) Intellectual ability and cortical development in children and adolescents. Nature 440: 676–679. 16572172 10.1038/nature04513

[7] Thompson DK, Wood SJ, Doyle LW, Warfield SK, Egan GF, et al (2009) MR-determined hippocampal asymmetry in full-term and preterm neonates. Hippocampus 19: 118–123. 18767066 10.1002/hipo.20492 PMC2631622

[8] Saitoh O, Karns CM, Courchesne E (2001) Development of the hippocampal formation from 2 to 42 years: MRI evidence of smaller area dentata in autism. Brain 124: 1317–1324. 11408327 10.1093/brain/124.7.1317

[9] Matsuzawa J, Matsui M, Konishi T, Noguchi K, Gur RC, et al (2001) Age-related volumetric changes of brain gray and white matter in healthy infants and children. Cereb Cortex 11: 335–342. 11278196 10.1093/cercor/11.4.335

[10] Almli CR, Rivkin MJ, McKinstry RC (2007) The NIH MRI study of normal brain development (Objective-2): newborns, infants, toddlers, and preschoolers. Neuroimage 35: 308–325. 17239623 10.1016/j.neuroimage.2006.08.058

[11] Schumann CM, Hamstra J, Goodlin-Jones BL, Lotspeich LJ, Kwon H, et al (2004) The amygdala is enlarged in children but not adolescents with autism; the hippocampus is enlarged at all ages. J Neurosci 24: 6392–6401. 15254095 10.1523/JNEUROSCI.1297-04.2004 PMC6729537

[12] Mosconi MW, Cody-Hazlett H, Poe MD, Gerig G, Gimpel-Smith R, et al (2009) Longitudinal study of amygdala volume and joint attention in 2- to 4-year-old children with autism. Arch Gen Psychiatry 66: 509–516. 19414710 10.1001/archgenpsychiatry.2009.19 PMC3156446

[13] Brenhouse HC, Andersen SL (2011) Developmental trajectories during adolescence in males and females: a cross-species understanding of underlying brain changes. Neurosci Biobehav Rev 35: 1687–1703. 21600919 10.1016/j.neubiorev.2011.04.013 PMC3134153

[14] Knickmeyer RC, Gouttard S, Kang C, Evans D, Wilber K, et al (2008) A structural MRI study of human brain development from birth to 2 years. J Neurosci 28: 12176–12182. 19020011 10.1523/JNEUROSCI.3479-08.2008 PMC2884385

[15] Gould E, Woolley CS, Frankfurt M, McEwen BS (1990) Gonadal steroids regulate dendritic spine density in hippocampal pyramidal cells in adulthood. J Neurosci 10: 1286–1291. 2329377 10.1523/JNEUROSCI.10-04-01286.1990 PMC6570209

[16] Martini L, Melcangi RC (1991) Androgen metabolism in the brain. J Steroid Biochem Mol Biol 39: 819–828. 1954172 10.1016/0960-0760(91)90031-y

[17] Lombardo MV, Ashwin E, Auyeung B, Chakrabarti B, Taylor K, et al (2012) Fetal testosterone influences sexually dimorphic gray matter in the human brain. J Neurosci 32: 674–680. 22238103 10.1523/JNEUROSCI.4389-11.2012 PMC3306238

[18] Buss C, Davis EP, Shahbaba B, Pruessner JC, Head K, et al (2012) Maternal cortisol over the course of pregnancy and subsequent child amygdala and hippocampus volumes and affective problems. Proc Natl Acad Sci U S A 109: E1312–1319. 22529357 10.1073/pnas.1201295109 PMC3356611

[19] Neufang S, Specht K, Hausmann M, Gunturkun O, Herpertz-Dahlmann B, et al (2009) Sex differences and the impact of steroid hormones on the developing human brain. Cereb Cortex 19: 464–473. 18550597 10.1093/cercor/bhn100

[20] Bramen JE, Hranilovich JA, Dahl RE, Forbes EE, Chen J, et al (2011) Puberty influences medial temporal lobe and cortical gray matter maturation differently in boys than girls matched for sexual maturity. Cereb Cortex 21: 636–646. 20713504 10.1093/cercor/bhq137 PMC3041011

[21] Toga AW, Thompson PM (2003) Mapping brain asymmetry. Nat Rev Neurosci 4: 37–48. 12511860 10.1038/nrn1009

[22] Whitwell JL, Crum WR, Watt HC, Fox NC (2001) Normalization of cerebral volumes by use of intracranial volume: implications for longitudinal quantitative MR imaging. AJNR Am J Neuroradiol 22: 1483–1489. 11559495 PMC7974589

[23] Raz N, Lindenberger U, Rodrigue KM, Kennedy KM, Head D, et al (2005) Regional brain changes in aging healthy adults: general trends, individual differences and modifiers. Cereb Cortex 15: 1676–1689. 15703252 10.1093/cercor/bhi044

[24] Fair DA, Dosenbach NU, Church JA, Cohen AL, Brahmbhatt S, et al (2007) Development of distinct control networks through segregation and integration. Proc Natl Acad Sci U S A 104: 13507–13512. 17679691 10.1073/pnas.0705843104 PMC1940033

[25] Gogtay N, Nugent TF 3rd, Herman DH, Ordonez A, Greenstein D, et al (2006) Dynamic mapping of normal human hippocampal development. Hippocampus 16: 664–672. 16826559 10.1002/hipo.20193

[26] Suzuki M, Hagino H, Nohara S, Zhou SY, Kawasaki Y, et al (2005) Male-specific volume expansion of the human hippocampus during adolescence. Cereb Cortex 15: 187–193. 15238436 10.1093/cercor/bhh121

[27] Benes FM (1989) Myelination of cortical-hippocampal relays during late adolescence. Schizophr Bull 15: 585–593. 2623440 10.1093/schbul/15.4.585

[28] van Praag H, Shubert T, Zhao C, Gage FH (2005) Exercise enhances learning and hippocampal neurogenesis in aged mice. J Neurosci 25: 8680–8685. 16177036 10.1523/JNEUROSCI.1731-05.2005 PMC1360197

[29] Payne C, Machado CJ, Bliwise NG, Bachevalier J (2010) Maturation of the hippocampal formation and amygdala in Macaca mulatta: a volumetric magnetic resonance imaging study. Hippocampus 20: 922–935. 19739247 10.1002/hipo.20688 PMC2891665

[30] Clark AS, MacLusky NJ, Goldman-Rakic PS (1988) Androgen binding and metabolism in the cerebral cortex of the developing rhesus monkey. Endocrinology 123: 932–940. 3260856 10.1210/endo-123-2-932

[31] Adriano F, Caltagirone C, Spalletta G (2012) Hippocampal volume reduction in first-episode and chronic schizophrenia: a review and meta-analysis. Neuroscientist 18: 180–200. 21531988 10.1177/1073858410395147

[32] Munson J, Dawson G, Abbott R, Faja S, Webb SJ, et al (2006) Amygdalar volume and behavioral development in autism. Arch Gen Psychiatry 63: 686–693. 16754842 10.1001/archpsyc.63.6.686

[33] Juranek J, Filipek PA, Berenji GR, Modahl C, Osann K, et al (2006) Association between amygdala volume and anxiety level: magnetic resonance imaging (MRI) study in autistic children. J Child Neurol 21: 1051–1058. 17156697 10.1177/7010.2006.00237

[34] Phelps EA, O’Connor KJ, Gatenby JC, Gore JC, Grillon C, et al (2001) Activation of the left amygdala to a cognitive representation of fear. Nat Neurosci 4: 437–441. 11276236 10.1038/86110

[35] Hardee JE, Thompson JC, Puce A (2008) The left amygdala knows fear: laterality in the amygdala response to fearful eyes. Soc Cogn Affect Neurosci 3: 47–54. 19015094 10.1093/scan/nsn001 PMC2569817

[36] Vythilingam M, Heim C, Newport J, Miller AH, Anderson E, et al (2002) Childhood trauma associated with smaller hippocampal volume in women with major depression. Am J Psychiatry 159: 2072–2080. 12450959 10.1176/appi.ajp.159.12.2072 PMC3230324

[37] Geschwind N, Levitsky W (1968) Human brain: left-right asymmetries in temporal speech region. Science 161: 186–187. 5657070 10.1126/science.161.3837.186

